# Are rare plant species less resistant than common ones to herbivores? A multi‐plant species study using above‐ and below‐ground generalist herbivores

**DOI:** 10.1002/ece3.10482

**Published:** 2023-09-05

**Authors:** Sarah Bürli, Andreas Ensslin, Anne Kempel, Markus Fischer

**Affiliations:** ^1^ Botanical Garden of the University of Bern Bern Switzerland; ^2^ Institute of Plant Sciences University of Bern Bern Switzerland; ^3^ Faculty of Health and Environmental Sciences Auckland New Zealand; ^4^ Conservatory and Botanic Garden of the City of Geneva Chambésy Switzerland; ^5^ WSL Institute for Snow and Avalanche Research SLF Davos Switzerland; ^6^ Climate Change, Extremes and Natural Hazards in Alpine Regions Research Centre CERC Davos Switzerland

**Keywords:** apparency hypothesis, feeding experiment, generalist invertebrate herbivores, growth‐defence trade‐off, herbivore performance, herbivore preference, plant palatability, regional and local rarity

## Abstract

Rare plant species are suggested to be less resistant to herbivores than common species. Their lower apparency and the fact that they often live in isolated populations, resulting in fewer herbivore encounters, might have led to the evolution of reduced defences. Moreover, their frequent lower levels of genetic diversity compared with common species could negatively affect their resistance against enemies. However, the hypothesis that plant resistance depends on plant regional and local rarity, independently of habitat and competitive and growth strategy, lacks evidence. To test this hypothesis, we assessed the performance and preference of one belowground and three aboveground generalist invertebrate herbivores from different taxonomic groups as indicators of plant resistance. Herbivores were fed a total of 62 regionally and locally rare and common plant species from Switzerland. We accounted for differences in a plant's growth and competitive strategy and habitat resource availability. We found that regionally and locally rare and common plant species did not generally differ in their resistance to most generalist herbivores. However, one herbivore species even performed better and preferred locally and regionally common plant species over rarer ones, indicating that common species are not more resistant, but tend to be less resistant. We also found that all herbivore species consistently performed better on competitive and large plant species, although different herbivore species generally preferred and performed better on different plant species. The latter indicates that the use of generalist herbivores as indicators of plant‐resistance levels can be misleading. Synthesis: Our results show that rare plant species are not inherently less resistant than common ones to herbivores. Instead, our results suggest that the ability of plants to allocate resources away from defence towards enhancing their competitive ability might have allowed plants to tolerate herbivory, and to become locally and regionally common.

## Graphical abstract

In our multi‐herbivore and multi‐plant species experiment, we address the link between plant resistance to herbivores and their local and regional rarity by putting it into context with their life‐history traits, habitats and evolutionary history. We found that rare plant species are not less resistant to herbivores than common ones. Instead, the ability of plants to allocate resources away from defence towards a stronger competitive ability might have allowed plants to tolerate herbivory and to become common.

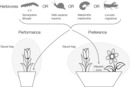

## 1 INTRODUCTION

Understanding why some plant species are locally or regionally rare (i.e. occur at low abundances or over a restricted range) while others are abundant and widespread has a long history in ecology with consequences for biodiversity management and conservation (Gaston, 1994). Hypotheses proposed to explain rarity usually focus on a plant's abiotic niche (Hanski, 1993; Slatyer et al., 2013). However, interactions with plant consumers have recently been also suggested to be potential drivers of local and regional plant rarity (e.g. Kempel et al., 2018; Klironomos, 2002). Hence, increased susceptibility of plants to consumers might restrict a species' ability to expand its range, or to become locally abundant. While evidence is accumulating that this might be the case for plant pathogens (Kempel et al., 2018; Mangan et al., 2010; Rutten et al., 2016; Xu et al., 2015; but see Reinhart et al., 2021), we lack knowledge of whether rare plant species are more susceptible to herbivores.

In addition, plant rarity itself might affect a species' susceptibility to herbivores. Plant species occurring at low abundances (i.e. locally rare) are less apparent and have a low probability to be found by herbivores (apparency hypothesis, Cates & Orians, 1975; Feeny, 1976; Rhoades & Cates, 1976). They are therefore suggested to allocate more resources to qualitative defences (e.g. alkaloids or glucosinolates), because they are low cost, as they are small molecules that are toxic at low doses. In comparison, apparent species, which are ‘bound to be found’ by herbivores, should allocate more resources to quantitative defences (e.g. phenols or tannins), which are costly and confer a broad‐spectrum defence (Feeny, 1976; Stamp, 2003). Additionally, regionally rare and threatened plant species often have declining populations and reduced genetic diversity (Ellstrand & Elam, 1993; Oostermeijer et al., 2003). This can negatively affect the formation of defences (Gaston, 1994; Spielman et al., 2004). Moreover, populations of regionally and locally rare species form ‘islands’ in the landscape (Feeny, 1976; Janzen, 1968), which leads to fewer encounters with herbivores (Altizer et al., 2007; Gibson et al., 2010; Smilanich et al., 2016). Such reduced exposure to herbivores may have led to the evolution of reduced herbivore defences in locally or regionally rare plant species. Hence, rare species may be more profitable to herbivores than common species (Laine, 2006) if they are subsequently exposed to herbivores. Some of these ideas have been tested in animals (Altizer et al., 2007) or with plant pathogens (Gibson et al., 2010; Kempel et al., 2018), but we still lack a general understanding of whether a plant's susceptibility to herbivores is related to a plant's regional and local rarity.

Plant susceptibility to herbivores also depends on a plant's growth and competitive strategy, and the environment in which a plant species has evolved (Kempel et al., 2020; Olff & Ritchie, 1998; Proulx & Mazumder, 1998). Plants from resource‐poor environments exhibit inherently slower growth rates than plants from resource‐rich environments (Coley et al., 1985). Consequently, slow‐growing species may be less able to replace lost tissue and invest more in defences than faster‐growing, more competitive species from productive environments, which typically tolerate herbivores better (growth‐defence trade‐off: Bryant et al., 1989; Coley et al., 1985; Díaz, 2000; Gianoli & Salgado‐Luarte, 2017; Grime, 1979). Moreover, many common species have adaptations for high resource acquisition and fast growth, whereas regionally rare species are characterised by resource conservatism and are thus limited to resource‐poor environments (Drury, 1974; Grime, 1979; Kempel et al., 2020). To rigorously test for differences in plant susceptibility to herbivory in regionally and locally rare and common plant species, it is therefore important to account for variation in plant growth and competitive strategy and the resource availability of the species' habitat.

The variety of defence and life strategies of plants (Coley et al., 1985; Karban & Baldwin, 2007; Walling, 2000), the host specificity of herbivores (Ali & Agrawal, 2012), their feeding strategies (Strong et al., 1984) and feeding compartments (above‐ or belowground) render the assessment of plant defence a complex endeavour, especially in a multi‐species framework. One approach to overcome this challenge is the use of generalist herbivores as indicators of plant resistance and susceptibility of plants to herbivores. According to Karban and Baldwin (2007)'s definition, a plant's resistance is a plant's response that reduce herbivore fitness (i.e. survival, performance and reproductive output) and preference. Unlike specialist herbivores, generalist herbivores feed on a variety of plant species, and respond strongly to variation in traits providing resistance to plants, such as nutritional quality and defensive compounds (often referred to as palatability; Kempel et al., 2015, 2018; Schädler et al., 2003). Their feeding response (performance) and preference can, therefore, serve as valuable tools for comparing plant resistance across numerous plant species. This becomes especially significant as these plants likely employ a myriad of diverse mechanical and chemical defences, making direct comparisons otherwise very difficult.

While such comparative feeding assays have been commonly used to inform about differences in defence investment and resistance between invasive and native plant populations (e.g. Caño et al., 2009; Siemann & Rogers, 2003) or species (e.g. Kempel et al., 2013; Pearson et al., 2011), they have rarely been used in the context of plant rarity, and if so, have involved only few plant species (Baskin et al., 1997; Cates & Orians, 1975; Fiedler, 1987; Landa & Rabinowitz, 1983; but see Ancheta & Heard, 2011; Cottam, 1985; Kempel et al., 2020). Moreover, studies rarely use different generalist herbivore species, investigate whether above‐ and belowground herbivores differ in their response to different plant species, or assess both herbivore performance and preference. Although herbivore performance and preference are expected to be tightly linked in herbivorous insects (‘mother‐knows‐best’ principle: Gripenberg et al., 2010; Jaenike, 1978), they may be targeted differentially by plant resistance (Kempel et al., 2015), and assessing both is therefore important.

Here, we present a multi‐species experiment where we compare the performance on 38 plant species and the preference on 56 plant species from Switzerland of one belowground and three aboveground generalist invertebrate herbivores from different taxonomic groups. We accounted for differences in plant regional and local rarity, plant's growth and competitive strategy and the resource availability of the species' habitat. Specifically, we addressed the following questions: (1) Do generalist herbivores differ in their performance and preference when feeding on locally and regionally rare plant species compared with common plant species? (2) Are the performance and preference of generalist herbivores affected by a plant's growth and competitive strategy, and resource availability of the species' habitat? (3) Are the herbivore performance and preference related within and across generalist herbivore species, and do different herbivore species perceive plant resistance similarly?

## 2 MATERIALS AND METHODS

### 2.1 Plant species and rarity

We selected 62 plant species from 16 families to cover a broad variety of families, rarity level, habitats and regions of Switzerland. Twenty species are common and 42 are rather rare to extremely rare in Switzerland (Table A1 in Appendix A). All rare species, except one (see explanation in Table A1 in Appendix A), have either a conservation priority (OFEV, 2019) or are near‐threatened or threatened (Swiss Red List of vascular plants; Bornand et al., 2016) in Switzerland.

As a measure of species regional rarity, we used the maximum range size of a species calculated as the highest number of 5 × 5 km grid squares that it occupied during the last century in Switzerland (Bornand, 2014). We used the range size in Switzerland, because the range size of the plant species used in this study is not yet available at this resolution at the European scale. However, Vincent et al. (2020) showed that European and Swiss range size of 21 plant species are positively correlated (*r* = .508, *p* < .001). As a measure of species local rarity, we used the indicator value for dominance in situ according to Landolt et al. (2010). This indicator value describes the accumulation of individuals of a plant species at the place where it occurs. It spans from a value of one for species with scattered individuals to five for species that are usually dominant.

### 2.2 Plant collection

Seeds of 10 seed families (i.e. from 10 different maternal plants) were collected from one or two populations of the 62 selected plant species. Rare and common plants were collected in the same regions of Switzerland. To break seed dormancy, Fabaceae seeds were scarified with a scalpel and seeds of other plant families were cold‐stratified in pots over 8 weeks in the dark at 4°C before they germinate in a greenhouse. After 8 weeks, 10 seedlings per seed‐family and population were randomly selected and pricked out individually into pots filled with a 1:9 mixture of sand and potting soil (Selmaterra). Plants were watered daily or every other day and allowed to grow for 3 months (constant day length of 14 h with additional light and temperature between 15 and 30°C).

### 2.3 Plant habitat, growth and competitive strategy traits

To characterise the resource availability of a species' habitat, we used the species indicator values for nutrients (N) and moisture (F) according to Landolt et al. (2010). Indicator values describe the realised ecological niche of a species by its position along an environmental gradient (Ellenberg et al., 1991; Landolt et al., 2010). The indicator values for nutrients and moisture indicate the nutrient content in the soil (mainly nitrogen) and the average soil moisture during the growth period of the species, following an ordinal scale ranging from 1 (very nutrient poor and very dry habitats) to 5 (very fertile and flooded habitats). They are the Swiss equivalent of the indicator values for nutrients and moisture according to Ellenberg et al. (1991). For each species, we also defined a variable called ‘competitive strategy’ on the basis of the species life‐strategies from Landolt et al. (2010; which was partly adapted from Grime's CSR life strategies; Grime, 1979, 2006). This variable describes the competitive ability for light of a species. To do so, we assigned the values ‘0’ to ruderal or stress‐tolerator (e.g. rrr, rrs, rss or sss), ‘1’ to competitive species (e.g. crr, csr or css) and ‘2’ to strongly competitive species (e.g. ccs or ccr; Table A1 in Appendix A). Plants with a high competitive ability and high nutrient and moisture indicator values may be more palatable to herbivores (Kempel et al., 2020; Olff & Ritchie, 1998; Proulx & Mazumder, 1998).

We measured the size of the plants as the highest stem height, or the longest leaf length including the petiole in the cases where the plants had only a rosette, and calculated the mean size per species. We measured the specific leaf area (here after called SLA), following the method from Cornelissen et al. (2003), and the chlorophyll concentration (chlorophyll concentration meter SPAD‐502 from Konica Minolta) of one leaf from five plants from different seed families per species. Then, we averaged the SLA and chlorophyll concentration per species. High SLA and high leaf chlorophyll concentrations are associated to a high plant palatability to herbivores (Coley & Barone, 1996; Poorter et al., 2004; Schuldt et al., 2012). SLA is a trait used in the leaf‐economic spectrum (Westoby, 1998; Wright et al., 2004), which distinguishes between plants with a fast growth strategy (species with short‐lived, nutrient‐rich leaves and high SLA) and plants with a slow growth strategy (species with long‐lived, nutrient‐poor leaves and low SLA). High SLA, nutrient requirement and competitive ability are known to be characteristics of fast‐growing plant species (Coley et al., 1985; Poorter & Remkes, 1990; Westoby, 1998). These traits are, therefore, regarded as key factors influencing plant resistance to herbivores, ultimately shaping the performance and preference of herbivores. Table A2 in Appendix A shows the mean and standard deviation of the plant variables for regionally and locally rare and common plant species.

### 2.4 Herbivore species

To assess herbivore performance and preference, we used one belowground and three aboveground invertebrate generalist herbivore species. As a belowground herbivore, we used larvae of the cockchafer *Melolontha melolontha* Linnaeus 1758 (hereafter called *Melolontha*; Coleoptera: *Scarabaeidae*). This species occurs naturally all over Europe (CABI, 2018) and has been a major pest in former times. Second instar *Melolontha* larvae were collected in agricultural fields in Urmein and Bristen in Eastern and Central Switzerland. None of our plant species were collected in these two regions, so we can rule out any potential pre‐adaptation of the herbivore to plant populations. Larvae were reared at 10°C in individual pots filled with a damp mix of grated carrots and soil. As aboveground herbivore, we chose the leaf‐chewing caterpillars of *Spodoptera littoralis* Boisduval 1833 (hereafter called *Spodoptera*; Lepidoptera: *Noctuidae*). This species occurs mostly not only in Africa and the Middle East but also in South Europe (CABI, 2018). Neonate caterpillars from laboratory‐reared strains (Syngenta) were kept at room temperature (24 ± 4°C) and fed ad libitum with maize‐based artificial diet until the start of the experiment. As further aboveground herbivore, we used *Helix aspersa maxima* snails Taylor 1883 (hereafter called *Helix*; Gastropoda: *Helicidae*). This species originates from North Africa but can be found nowadays in Europe, Asia, Australia, South and North America (CABI, 2018). Pre‐adult snails were bought from a commercial seller (Etis Schneckenpark). They were kept for 48 h in large plastic containers with 2 cm of damp soil and closed with plastic wrap to water‐saturate the air in the box and standardise snail water content (Ledergerber et al., 1998; Staikou, 1999) before they entered the experiment. During this time, they were fed ad libitum with fresh lettuce leaves for the first 24 h and then with wet paper tissues to standardise their gut content. As last aboveground herbivore, we used locusts of the species *Locusta migratoria* Linnaeus 1785 (hereafter called *Locusta*; Orthoptera: *Acrididae*). This species occurs naturally in Europe, Africa, Asia and Australia (CABI, 2018). Pre‐adult locusts were bought from a commercial seller (Pocerias) and placed in boxes with sawdust and fed with egg cartons to standardise their gut content for the last 24 h before they entered the experiment. All four herbivore species are adequate model organisms to study herbivore performance and preference on plants because they are known to feed on a wide range of plant species (Bernays & Chapman, 1977; Bont et al., 2017; Brown & Dewhurst, 1975; Gomot & Pihan, 1997; Kempel et al., 2015). Moreover, as the three non‐native herbivore species to Switzerland are unlikely to share a co‐evolutionary history with any of the plant species used in this study, it is expected that their fitness and behaviour reflects the general quality of the different plant species as food source.

### 2.5 Herbivore experiments

**FIGURE 1:**
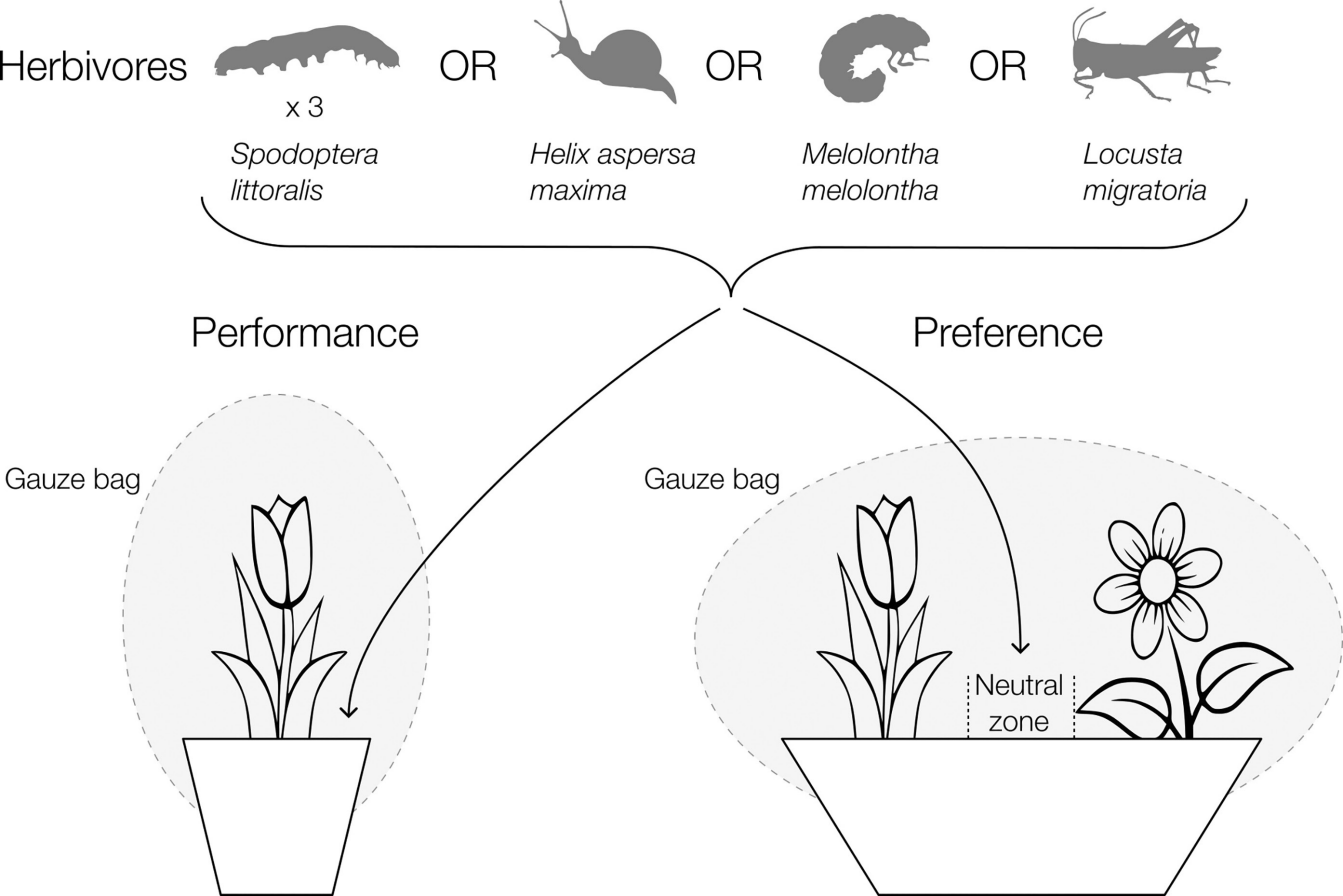
Experimental sketch. Sketch of the performance experiment (left) and the preference experiment (right) for four herbivore species.

For all herbivores, two experiments using multiple plant species were conducted in a greenhouse: a no‐choice feeding experiment, hereafter called Performance Experiment, and a pairwise choice experiment, hereafter called Preference Experiment (Figure 1).

#### 2.5.1 Performance experiment

We assessed the herbivore performance on 38 plant species from July to October 2018. For each herbivore species, plants of similar size from different seed families of 23–34 species were used (see summary of the experimental conditions in Table A3 in Appendix A). The number of plant species differed between herbivore species due to low germination and mortality between the different herbivore‐species experiments. Thus, some plants were used to assess the performance of more than one herbivore species. However, these cases were rare: Of the 1638 plants, 275 plants (17%; 106 common and 169 rare plants) were used twice and two plants (0.1%, one common and one rare plant) were used three times. We were able to rule out any bias due to resistance induced in plants by contact with previous herbivorous species (Karban & Baldwin, 2007; see Section 2.6 below).

For the experiments with *Melolontha*, *Helix* and *Locusta*, we added one larva, one snail and one locust to each plant, respectively. The experiment with *Spodoptera* was replicated twice, due to a high mortality rate of the caterpillars. We added two 4‐days‐old and three 1‐week‐old caterpillars to each plant for the first and the second experimental replication, respectively. Plants were then individually bagged with a gauze bag to prevent the escape of the herbivores. We allowed *Melolontha* to feed on the plants for 15 days, *Locusta* for 5 days, *Helix* for 7 days and *Spodoptera* for 10 and 5 days, respectively. We chose different feeding durations for the different herbivores because they differ in mobility, developmental stage, size and growth rates (Table A3 in Appendix A).

To quantify herbivore performance, we weighed the living herbivores to the nearest hundredth gram before and after the feeding (Cubis balance MSA225P‐100‐DI, Sartorius). For each of the two experimental replications with *Spodoptera*, we calculated the mean weight of the caterpillars per pot before and after the feeding. We considered only herbivores that did not run out of food (i.e. the plant was not entirely eaten) and were alive at the end of the feeding time. A herbivore might not gain weight either because the food may be of poor nutritious quality or because it suffers from food‐independent mortality or illness. As those two cases cannot be distinguished, we used only the data from herbivores gaining weight (16.6% of the herbivores did not gain weight and their removal did not result in the exclusion of plant species).

#### 2.5.2 Preference experiment

We assessed the preference of the herbivores to 56 plant species from June to July 2019. Seeds of additional plant species were collected for the preference experiment compared with the performance experiment. For each herbivore species, plants of similar size from different seed families of 49–55 species were selected (Table A3 in Appendix A). The number of plant species differed between herbivore species due low germination and mortality between the different herbivore‐species experiments. Thus, some plants were used to assess the preference of more than one herbivore species. Of the 1242 plants, 365 plants (29%) were used to assess the preference of more than one herbivore species. It is unlikely that possible induced resistance in plants due to the contact with previous herbivore species (Karban & Baldwin, 2007) have biased our results in the preference assessment of subsequent herbivores species, although we cannot fully rule this out, given that the data are aggregated to the plant species level (see below).

For each herbivore species, we conducted a series of pairwise choice tests, during which a herbivore could choose between two different plant species (Figure 1). The two plant species for each choice test were potted together, c. 10 cm apart, in a pot (i.e. experimental unit; 30 × 12 × 11 cm). One *Helix*, *Locusta*, *Melolontha* and three 10–12 days old *Spodoptera*, respectively, were used per experimental unit. Herbivores were positioned in the middle of the pot. Pots were bagged with a gauze bag. *Melolontha* were placed in a hole in the middle of the pot 24 h after the plants had been potted together to allow root exudates to mix in the soil.

We assessed the preference of *Helix*, *Melolontha* and *Spodoptera* after 24 h by counting the number of herbivores on each of the two plants. We refer to the number of herbivores as ‘goals’ in analogy to football. Herbivores that stayed in the middle of the pot between the two plants, that is, the ‘neutral zone’ (Figure 1), were not counted. As locusts are very mobile species, we recorded the position of the locust every 5 min for 30 min and summed up the number of goals per plant species (giving a maximum of six goals per plant species). Therefore, per experimental unit, a plant can get a maximum of three goals with *Spodoptera*, six goals with *Locusta* and one goal with *Helix* and *Melolontha*.

To reduce the prohibitively large number of tests that would have been required to test each possible pair of plant species ([*n*(*n*−1)]/2 tests, where *n* is the number of plant species; 1540 tests for 56 plant species), we did two rounds of round‐robin tests (Kempel et al., 2015). We randomly assigned plant species to eight groups of six to seven species in the first round and assessed herbivore preference for each possible combination of species within each group. For the second round, we formed seven new groups of seven to eight plant species according to the preference ranking of the species within their group. Hence, groups in the second round were equally powerful, as they each contained one species from each rank and group of the first round. Then, we assessed again herbivore preference for each possible combination of species within each group. Each plant species had the same number of tests and played against preferred and less preferred species. A total number of 338, 273, 351 and 357 tests were performed to assess the preference of *Helix*, *Locusta*, *Melolontha* and *Spodoptera*, respectively. The number of tests per herbivore species varied in function of the number of available healthy herbivores and plants. Finally, we summed up the number of goals per plant species for all tests and both rounds and used this as an indicator of herbivore preference. Plant species with many goals were considered the most preferred by herbivores, whereas plant species with very few goals were considered the least preferred.

### 2.6 Statistical analysis

To test whether plant species of different regional and local rarity, growth and competitive strategy and habitat differed in their resistance to generalist herbivores, we fitted two linear mixed‐effect models in R 3.5.3 (R Core Team, 2019). For inference scale of our analyses, please refer to Table 1. Including the four herbivore species together, one model was fitted for herbivore performance and one for preference (lme4, lmerTest and MuMIn R packages; Barton, 2020; Bates et al., 2015; Kuznetsova et al., 2017).

**TABLE 1:** Inference table.

Scale of inference	Scale at which the factor of interest is applied	Number of replicates at the appropriate scale
Plant species	Plant species	Thirty‐eight and 56 plant species for the performance and preference experiments, respectively, differing in regional and local rarity, growth and competitive strategy and habitat
Herbivore species	Herbivore species	Four herbivore species

Response variables of the performance and preference model were the final weight of the herbivores (or the mean final weight per experiment for *Spodoptera*) and the number of goals that herbivore species awarded to plant species, respectively. In the performance model, we included the initial weight of the herbivore and its interaction with herbivore species as a covariate to correct for herbivore‐specific initial weight and herbivore species‐specific weight gain. We also fitted the performance model with weight difference and weight ratio. However, based on the residual distribution, *R* ^2^ and AIC, we opted for the former model. The initial weight and both response variables were centred and scaled per herbivore species to standardise weight and goal number between herbivore species. As random effects, we included plant seed families nested in plant species for the performance model and plant species for the preference model.

**FIGURE A1:**
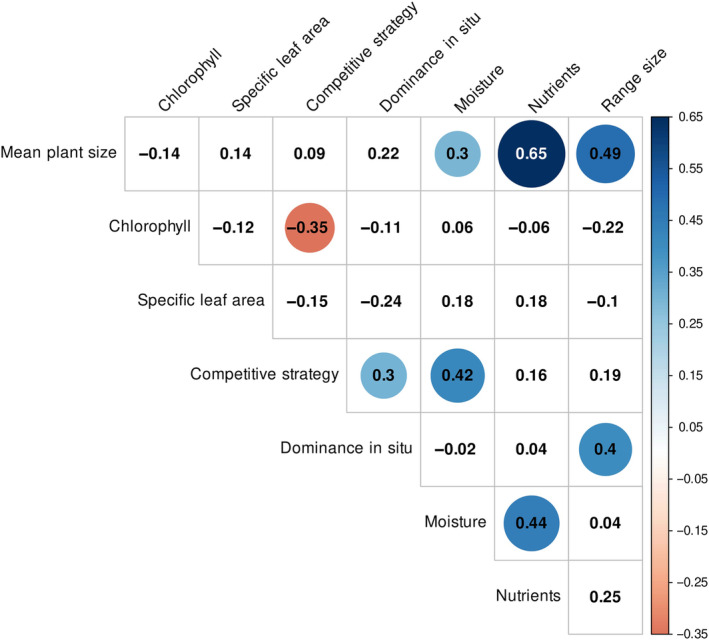
Correlation between plant variables. Pearson correlation coefficients between the plant traits, strategy, indicator values and range size. Blue and red circles correspond to positive and negative significant (*p* < .05) correlations, respectively.

We included the plant species range size (log transformed), indicator values for the dominance in‐situ, nutrients and moisture, competitive strategy, SLA, leaf chlorophyll concentration (all numeric), herbivore species and all two‐way interactions as fixed effects in the models (Table A4 in Appendix A). We treated the competitive strategy and the three Landolt indicator values as numeric variables, since they reflect ecological gradients (see also Boch et al., 2019; Bornand, 2014) and to facilitate the inference and generalisation of our results to plant species situated at more extreme values along these gradients. To correct for potential effects of within plant‐species variation in plant volume on herbivore performance or preference, we included the initial plant size in the performance model and the initial mean size per plant species in the preference model as additional covariates. Since no pair of highly multi‐collinear variables was found (*r* > .7; Figure A1 in Appendix A), all variables were retained in both models. Numeric covariates and explanatory variables were centred and scaled. We reduced the models by using a backward stepwise procedure to remove the least significant terms (Table A4 in Appendix A; significance threshold: *p* < .05) calculated with the Satterthwaite's method of approximation (Kuznetsova et al., 2017). A backward stepwise procedure allowed us to simultaneously examine the effects of all the variables for which we have hypothesised an effect on herbivore performance and preference, and then to identify those that are actually important (i.e. significant in the model).

To check for the effect of possible induced resistance (Karban & Baldwin, 2007) in plants used to assess the performance of more than one herbivore species performance, we removed these plants from the data set and proceeded to the model reduction. Because the results were qualitatively the same, we kept these plants in the model. In addition, we ran models for each herbivore species separately to calculate herbivore‐specific regressions for any significant plant variables. In this case, herbivore species was removed from the fixed terms and we used a linear mixed‐effect model for the performance and a linear model for the preference. We also tested whether a herbivore‐species performance was related to its preference. To do that, we extracted the residuals from the linear model of the herbivore final weight in function of the initial weight and ran a linear model where the residuals were explained by the herbivore preference. We also calculated the Pearson correlation between the performance and preference within and between herbivore species. Graphs were performed with R packages ggplot2, corrplot, effects and remef (Fox & Weisberg, 2018; Hohenstein & Kliegl, 2020; Wei & Simko, 2017; Wickham, 2016).

To test whether herbivore performance and preference were related to plant phylogeny and whether closely related plant species share similar range size, indicator values, traits and strategy, we constructed a plant‐species phylogenetic tree using the dated plant phylogeny of Smith and Brown (2018). We then tested for a phylogenetic signal in the preference and performance of each herbivore species and in each plant variable (Blomberg et al., 2003; phytools R‐package, Revell, 2012). As none of the variables presented a significant phylogenetic signal (Table A5 in Appendix A), analyses with a phylogenetic correction were not considered necessary (Carvalho et al., 2006).

## 3 RESULTS

### 3.1 Performance experiment

**FIGURE 2:**
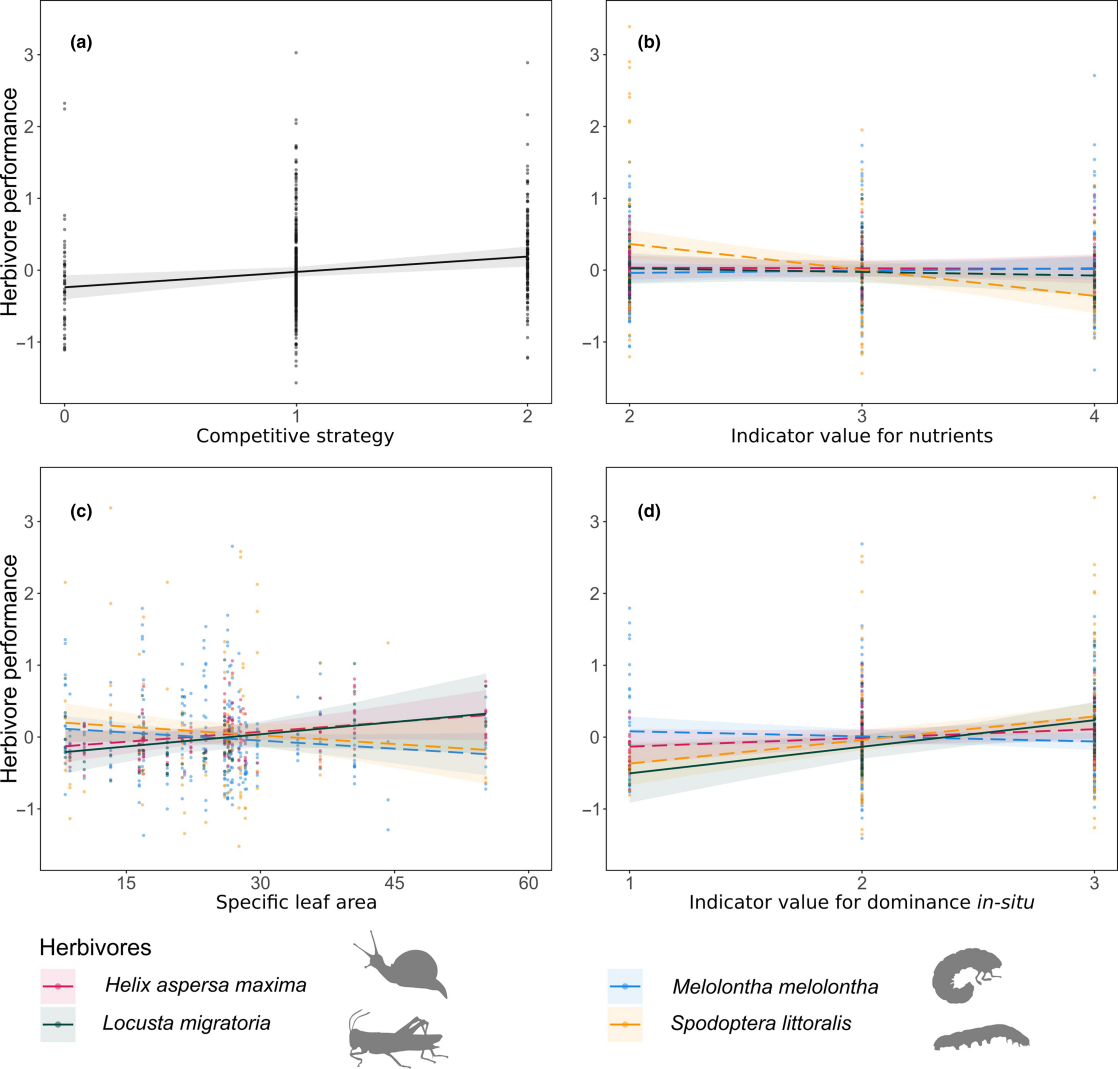
Herbivore performance. Relationship between herbivore performance (model estimates) and (a) plant competitive strategy for all herbivores together, (b) plant indicator value for nutrients, (c) SLA and (d) plant indicator value for dominance in situ per herbivore species. Points show partial residuals. Solid and dashed lines indicate significant (*p* < .05) and non‐significant regression lines, respectively.

All four herbivore species performed better on competitive plant species (Figure 2a, Table 2). Indeed, all herbivores gained more weight when feeding on competitive plant species compared to non‐competitive species. Herbivore performance was also related to the plant SLA and indicator value for nutrients and dominance in situ, however, the effects varied significantly between herbivore species (Figure 2b–d, Table 2). Herbivore performance was not related to the plant range size, indicator value for moisture and leaf‐chlorophyll concentration, nor did the initial plant size affect the performance of the herbivores.

**TABLE 2:** Performance model for all herbivore species.

Performance—All herbivores				
Fixed effects	Sum Sq	df	*F*	*p*
Initial plant size	0.899	1	2.677	.106
Herbivore initial weight	210.030	1	625.53	**<.001**
Herbivore species	0.501	3	0.498	.684
Herbivore species × Herbivore initial weight	49.171	3	48.815	**<.001**
N	1.004	1	2.991	.092
Competitive strategy	3.167	1	9.431	**.005**
SLA	0.030	1	0.089	.768
Dominance	2.862	1	8.523	**.006**
N × Herbivore species	6.705	3	6.656	**<.001**
SLA × Herbivore species	3.554	3	3.528	**.015**
Dominance × Herbivore species	5.950	3	5.907	**<.001**

Random effects	Variance	df	LRT	*p*
Seed family	0.000	1	0.00	1
Plant species	0.016	1	5.392	**.02**
Residuals	0.358			

*Note*: Results of linear mixed effect model testing for the effect of plant traits and strategy, indicator values and range size on the performance of the herbivore species together after model reduction. Note that the range size, chlorophyll concentration and indicator value for moisture were not retained in the final model, as they were not significant. Performance analysis was conducted on 610 observations, 212 seed families and 38 plant species. Sum Sq, df, *F*, *p* and LRT refer to the sum of squares, degrees of freedom, *F*‐statistic value, corresponding *p* value and log‐likelihood ratio test statistic, respectively. Significant *p* values are highlighted in bold.

In detail, *Spodoptera* performance tended to decrease on plants from nutrient‐rich habitats, while the performance of the other herbivores was not affected by a plant's nutrient indicator value. The performance of the herbivores in response to SLA differed between herbivore species: *Helix* and *Locusta* performed better on high‐SLA species, while *Melolontha* and *Spodoptera* performed better on low‐SLA species (Figure 2c). However, only the performance of *Locusta* varied significantly with SLA: This herbivore performed significantly better on high‐SLA plant species (Table A6 in Appendix A). Because SLA is a proxy of plant growth strategy, where species with high SLA usually grow faster and are more palatable, this result indicates that the performance of *Locusta* is affected by the quality of the plant as food. *Spodoptera* and *Locusta* performances increased with plant dominance, while the performance of *Helix* and *Melolontha* was not affected by it (Figure 2b,d). However, only the performance of *Locusta* varied significantly with plant dominance: *Locusta* performed better on dominant plant species than on less dominant species. This result indicates that plant species with a high local abundance possess traits that increase the performance of this generalist herbivore species.

### 3.2 Preference experiment

**FIGURE 3:**
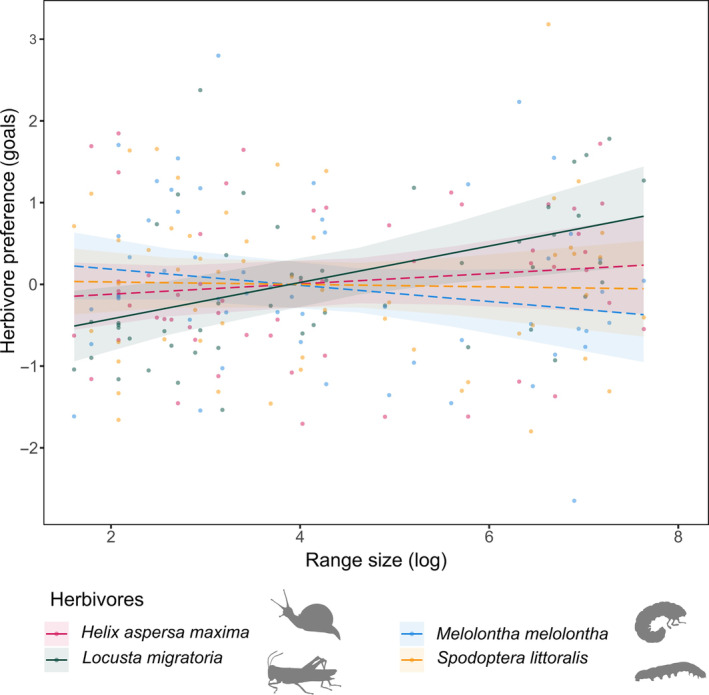
Herbivore preference. Relationship between the herbivore preference (model estimates based on the number of goals) and plant range size (log transformed). Points show the partial residuals. Solid and dashed lines indicate significant (*p* < .05) and non‐significant regression lines, respectively.

The preference of the herbivores was related to the plant range size in a herbivore species‐specific way (Figure 3, Table 3). *Locusta* significantly preferred regionally common plant species compared to regionally rare species, while the preference of the other herbivore species was not affected by range size (Figure 3, Table A7 in Appendix A). The initial mean plant size, which we used as a covariate, also affected the preference of all four herbivores, with larger plant species being preferred over smaller ones. Herbivore preference was not related to SLA, leaf‐chlorophyll concentration and any of the indicator values of the plant species.

**TABLE 3:** Preference model for all herbivore species.

PREFERENCE—All herbivores				
Fixed effects	Sum Sq	df	*F*	*p*
Initial mean plant size	6.972	1	8.176	**.006**
Herbivore species	0.001	3	0.000	1
Range size (log)	0.87	1	1.020	.317
Range size (log) × Herbivore species	10.288	3	4.022	**.009**

Random effects	Variance	df	LRT	*p*
Plant species	0.042	1	0.647	.421
Residuals	0.853			

*Note*: Results of linear mixed effect model testing for the effect of plant traits, indicator values and range size on the preference of the herbivore species together after model reduction. Note that the SLA, chlorophyll concentration, competitive strategy, indicator values for nutrients, moisture and dominance in situ were not retained in the final model, as they were not significant. Preference analysis was conducted on 213 observations and 56 plant species. Sum Sq, df, *F*, *p* and LRT refer to the sum of squares, degrees of freedom, *F*‐statistic value, corresponding *p* value and log‐likelihood ratio test statistic, respectively. Significant *p* values are highlighted in bold.

**FIGURE 4:**
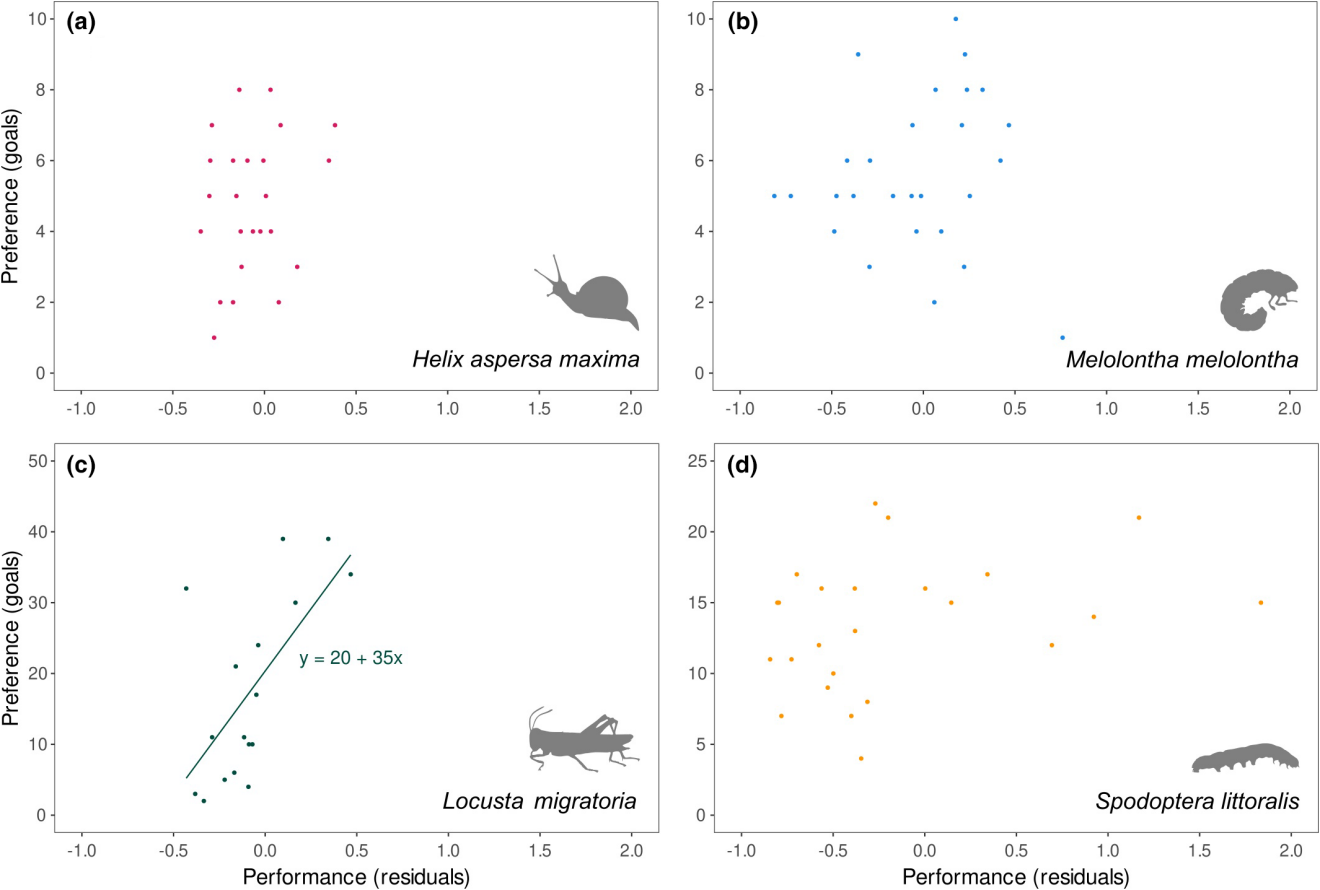
Relationship between herbivore preference and herbivore performance for (a) Helix, (b) Melolontha, (c) Locusta and (d) Spodoptera.

**FIGURE A2:**
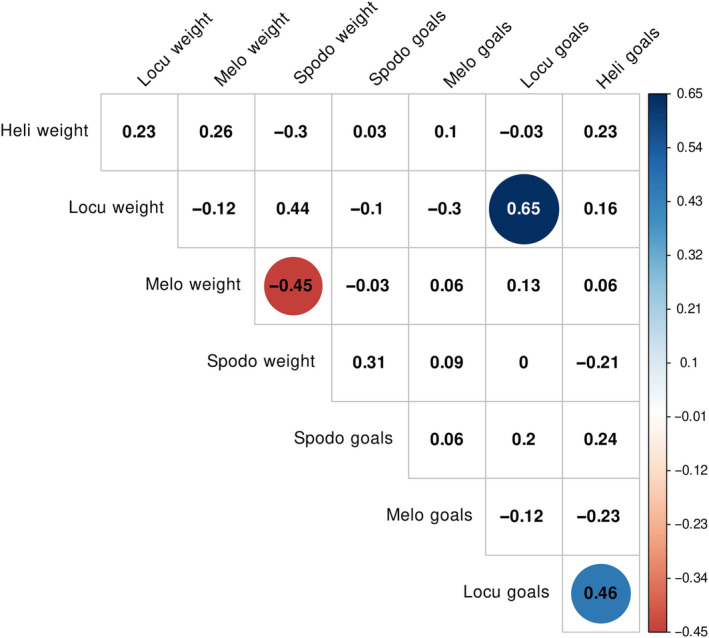
Correlation between herbivore performance and preference. Pearson correlation coefficients between herbivore performance (final herbivore weight adjusted for initial weight) and preference (i.e. number of goals). Blue and red circles correspond to positive and negative significant (*p* < .05) correlations, respectively. Heli, Locu, Melo and Spodo refer to *Helix aspersa maxima*, *Locusta migratoria*, *Melolontha melolontha* and *Spodoptera littoralis*, respectively.

The herbivore preference was generally independent of herbivore performance within and across herbivore species with the exception of the preference and performance of *Locusta* (*r* > .6; linear model: df = 1, Sum of squares = 1150.0, *F* = 10.875, *p* = .005), the preference of *Helix* and *Locusta* and the performance of *Melolontha* and *Spodoptera* that were slightly correlated (*r* ≃ .4, *p* < .05; Figure 4, Figure A2 in Appendix A).

## 4 DISCUSSION

### 4.1 Common plant species are not more resistant against generalist herbivores than rare plant species

Our results indicate that three generalist herbivore species out of four did not differ in their performance and preference when feeding on regionally and locally rare plant species compared with common and dominant plant species. We found these results after accounting for plant habitat, traits and growth strategy, which are factors known to alter a plant's susceptibility to herbivores. Hence, our results suggest that regionally and locally rare and common plant species do not differ in their resistance against most generalist herbivores. This is in line with Kempel et al. (2020), who found no difference in ambient herbivory between 40 regionally rare and common plant species. However, the few other studies that investigated the relationship between plant rarity and susceptibility to herbivory found contrasting results. Landa and Rabinowitz (1983) showed that a generalist grasshopper preferred regionally rare over common grasses (seven species). Similarly, Kempel et al. (2018; 19 species) reported that regionally rare species but not locally rare species were more susceptible to soil biota than common plant species, and Fiedler (1987; four species) found that regionally rare species were more susceptible to leaf herbivory. In contrast, Leege et al. (2010) found that two regionally common *Trillium* species tended to be more susceptible to herbivory than a rare *Trillium* in common gardens. Thus, studies based on few species and not accounting for differences in plant growth strategies may provide a biased view of the relationship between plant rarity and resistance to herbivory.

In our study, only one herbivore species, *Locusta*, distinguished between locally rare and common and between regionally rare and common plant species. This herbivore had a higher performance on locally common (i.e. dominant) plant species and preferred regionally common over rare plant species. This result suggests that if, as hypothesised by the apparency hypothesis (Feeny, 1976), locally common plant species invest more into quantitative defences, which confer a broad‐spectrum defence to the plant, than locally rare plant species, these defences do not confer a plant resistance against *Locusta*. This results also contrasts with the idea that rare and threatened plant species with declining, isolated populations are less resistant to herbivory due to lower genetic diversity (Spielman et al., 2004) or due to the evolution of reduced defence because of lower herbivore exposure (Altizer et al., 2007; Gibson et al., 2010; Laine, 2006). Our results with *Locusta* indicate that locally and regionally common species are less, rather than more, resistant to herbivory. Furthermore, it was observed that all herbivores showed a preference for larger plants over shorter ones, aligning with the apparency hypothesis, which proposes that larger plants are more apparent and appealing to herbivores as a resource. Surprisingly, despite their higher attractiveness, larger plants did not exhibit increased resistance (i.e. reduced performance) to herbivores. A possible explanation might be that only a low investment to defence allows plants to become locally abundant and large in size, given that it is commonly assumed that defence is costly and trades off with growth (Coley et al., 1985; Züst & Agrawal, 2017). Such fast‐growing, competitive species are often more tolerant to herbivores than less abundant species, as they can easily replace lost biomass (Gianoli & Salgado‐Luarte, 2017). Similarly, fast‐growing species and species more tolerant to generalist herbivores may be better able to spread and become regionally common, particularly if they are more limited by competing neighbouring plants than by herbivores. Hence, our multi‐plant and herbivore‐species experiment challenges the idea that common plant species are more resistant to herbivores than rare plant species. On the contrary, common plant species tend to be less resistant than rarer species. The ability to allocate resources away from defence to vigorous growth might therefore be important for plants to become locally and regionally common but might come with the cost of a higher susceptibility to herbivores.

### 4.2 Plant competitive ability is related to plant resistance and palatability

Generalist herbivores performed better on competitive plant species in our study. Competitors according to Grime's CSR life‐strategy scheme (Grime, 1974, 2006) are plant species with fast resource acquisition in productive environments, short‐lived leaves and high allocation to vegetative growth (Grime & Pierce, 2012). These traits are also tightly linked to a plant's strategy against herbivores (Kempel et al., 2015; Kuglerová et al., 2019; Poorter & Remkes, 1990; Rosenthal & Kotanen, 1994), as plant growth is typically suggested to trade‐off with plant defence (Cappelli et al., 2020; Coley et al., 1985; Endara & Coley, 2011). Our results thus indicate that highly competitive plant species invest less into resistance against herbivores than less competitive species, which supports the idea of a growth‐defence trade‐off.

Other plant traits investigated in this study were not consistently related to herbivore performance. The nutrient availability of a plant's habitat and SLA were related to herbivore performance, but their effects varied considerably across herbivore species. Plant traits were also weakly correlated with each other. This indicates that most single plant traits are limited in explaining herbivore responses, as individual traits evaluate only one aspect of a plant's palatability and different herbivore species respond differently to them as they differ in anatomy, morphology and physiology. This also explains why studies reported contrasting relationships between herbivory and plant palatability when using single traits (e.g. compare Knepp et al., 2005; Kuglerová et al., 2019; Lamarre et al., 2012; Loranger et al., 2012; Moles & Westoby, 2000; Schädler et al., 2003). A combination of traits is likely to better predict herbivore responses, which is also supported by the concept of plant defence syndromes (Agrawal & Fishbein, 2006). The fact that Grime's CSR scheme consistently explains herbivore performance in our study suggests that trait combinations representing the two axis of the plant‐economic spectrum (Díaz et al., 2016), such as SLA and plant height, might be particularly promising candidates for predicting plant palatability. Nevertheless, future studies combining different plant traits, including qualitative and quantitative defence traits, could provide further insights into the relationship between plant growth and resistance against generalist but also specialist herbivores, as they were reported to differ in their sensitivity to those defence types (Rhoades & Cates, 1976).

### 4.3 Herbivore preference is not consistently related to performance

The relationship between preference and performance in insect herbivores is suggested to be tightly linked, since females are under strong selective pressure to oviposit on plant species that maximise offspring fitness (‘mother‐knows‐best’: Gripenberg et al., 2010; Jaenike, 1978). Although the ‘mother‐knows‐best’ principle applies to insects across two generations, Kempel et al. (2015) reported that herbivore performance and preference for plant species are correlated within the same generation. In our study, herbivore preference and performance were only related in *Locusta*. Moreover, different herbivore species seem to perceive plant resistance differently, as the performance and preference between herbivore species were rarely correlated. The absence of correlation between *Spodoptera* performance and preference in our study may be due to a slight age difference between the caterpillars used in the performance and the preference experiment (Table A3 in Appendix A). Caterpillars may have differed in their mandible development and thus their ability in feeding on less palatable leaves. However, independence of herbivore preference and performance was also reported in other studies (Bernays, 1990; Cronin & Abrahamson, 2001; Duffy & Hay, 1991). In these cases, it was suggested that herbivores had chosen plant species on the basis of highly conservative and simple cues (e.g. taste) or traits unrelated to plant quality as food, such as a plant's suitability for providing shelter. The later reason seems likely, as the preference of all four herbivores was significantly related to the initial mean size of the plant species in our study (Table 3). The absence of phylogenetic signal in herbivore performance and preference also suggests that preference and performance of herbivores used in this study is not determined by secondary compounds, which are often evolutionary conserved, but rather by other cues.

Our results contrast with two common ideas in ecology: (1) herbivore performance and preference are tightly linked (Gripenberg et al., 2010) and (2) food plant quality is recognised in a similar way by different generalist herbivore species (Herms & Mattson, 1992; Pérez‐Harguindeguy et al., 2003). A reason why plant quality was perceived differently by most herbivore species used in our study might be that all herbivores originate from different taxonomic groups or even inhabit different compartments (above‐ and belowground). *Locusta migratoria* is known to have a preference for grasses (Ohabuike, 1979), while gastropods such as *Helix* prefer herbs (Iglesias & Castillejo, 1999 and the references herein). In addition, orthopterans are known for dietary mixing (Bernays & Bright, 1993; Unsicker et al., 2008), potentially masking their performance and preference in laboratory feeding trials. Further, living conditions of soil herbivores differ greatly from those aboveground, questioning that mechanisms driving food preference aboveground are the same in the soil, where the movement and perception of plant signals are impeded (Schallhart et al., 2012). Although these issues might explain our results, they also raise questions about the suitability of a single generalist herbivore species as indicator of plant resistance. Generalist herbivores are diverse, and although preferences for certain plant species might correlate within certain taxonomic groups, plant quality seems to be largely differently perceived by herbivores differing in feeding types or living compartment.

## 5 CONCLUSION

Our multi‐species experiment suggests that regionally and locally rare plant species are not less resistant to generalist herbivores than regionally and locally common plant species. Instead, there are indications that common species are slightly less resistant to generalist herbivores than rare species. This is possibly because common species allocate more resources to a vigour growth, which allows them to form dense patches and to become locally and regionally common. In line with this idea, we found that competitive plant species are more palatable to generalist herbivores than are ruderal or stress‐tolerator species. It is possible that their low investment in defence allows such plant species to become successful in productive environments. We conclude that plant rarity is related to herbivore resistance in herbaceous plant species only in a very herbivore species‐specific way. Rather, it is the competitive strategy and the allocation of resources to vigorous growth that drive general patterns of resistance to generalist herbivores in plants.

## AUTHOR CONTRIBUTIONS

**Sarah Bürli:** Conceptualization (equal); data curation (lead); formal analysis (lead); investigation (lead); methodology (equal); writing – original draft (lead); writing – review and editing (equal). **Andreas Ensslin:** Conceptualization (equal); methodology (equal); writing – review and editing (equal). **Anne Kempel:** Conceptualization (equal); methodology (equal); writing – review and editing (equal). **Markus Fischer:** Conceptualization (equal); funding acquisition (lead); methodology (equal); writing – review and editing (equal).

## CONFLICT OF INTEREST STATEMENT

The authors declare no conflicts of interest.

## Data Availability

Data are archived on the international open‐access repository DRYAD: Bürli et al. (2023).

## APPENDIX A

**TABLE A1:** Plant species list.

Plant species	Rarity	Perf	Pref	Plant family	Plant size	Chloro	SLA	Comp. strat.	F	N	D	Range size
*Achillea millefolium*	Common	×	×	Asteraceae	37	6.5	8.2	2	2	3	3	2070
*Aira elegantissima*	Rare	×		Poaceae	18.5	NA	NA	0	1	1	1	3
*Androsace septentrionalis*	Rare		×	Primulaceae	6.7	56.6	20.5	0	1	3	2	15
*Arabis turrita*	Common	×	×	Brassicaceae	37	15.7	40.5	1	2	2	2	270
*Arenaria grandiflora*	Rare	×	×	Caryophyllaceae	12.1	4.5	55.2	1	2	2	1	8
*Artemisia borealis*	Rare	×	×	Asteraceae	16	11.8	13.2	1	2	2	3	11
*Artemisia glacialis*	Rare	×		Asteraceae	6.2	NA	NA	1	2	2	2	10
*Bidens cernua*	Rare		×	Asteraceae	28.8	32.2	56.9	1	5	4	1	56
*Bromus squarrosus*	Rare	×	×	Poaceae	58.9	43.9	34.2	1	2	4	3	55
*Campanula cervicaria*	Rare		×	Campanulaceae	27.1	32	30.2	1	3	3	1	25
*Carpesium cernuum*	Rare	×	×	Asteraceae	36.9	31.1	26.3	1	3	4	1	15
*Caucalis platycarpos*	Rare	×		Apiaceae	27.6	NA	NA	0	2	3	1	51
*Centaurea jacea*	Common	×	×	Asteraceae	47.1	34.1	26	1	3	3	3	1128
*Centaurea nigra*	Rare		×	Asteraceae	27.8	37.5	31.7	1	3	3	3	30
*Centaurea valesiaca*	Rare	×	×	Asteraceae	44	22.1	18.6	1	1	2	3	40
*Chenopodium album*	Common		×	Chenopodiaceae	72.5	51	19.8	0	2	4	2	810
*Cicuta virosa*	Rare		×	Apiaceae	56.4	22.6	25.9	1	5	3	1	23
*Cochlearia pyrenaica*	Rare	×	×	Brassicaceae	10.2	59.6	10.3	1	5	2	3	8
*Crepis froelichiana*	Rare	×	×	Asteraceae	7.5	43.8	22.2	1	3	2	1	5
*Dactylis glomerata*	Common	×	×	Poaceae	82.1	23.9	26.7	1	3	4	3	1438
*Erysimum cheiranthoides*	Common	×	×	Brassicaceae	72.1	56.4	28.5	0	4	4	2	140
*Erysimum ochroleucum*	Rare		×	Brassicaceae	23.9	26	27.3	1	1	2	2	6
*Genista pilosa*	Rare		×	Fabaceae	14.3	28.9	22.7	2	2	1	3	23
*Hypericum perforatum*	Common	×	×	Hypericaceae	64.1	14.3	28.3	1	3	3	3	1115
*Hypericum richeri*	Rare	×	×	Hypericaceae	43.3	37.1	26.4	1	3	3	2	24
*Hypochaeris radicata*	Common	×	×	Asteraceae	30.4	39.1	23.7	1	3	2	2	957
*Knautia arvensis*	Common	×	×	Dipsacaceae	44.7	35.5	21.3	2	3	3	2	1039
*Laserpitium siler*	Common		×	Apiaceae	21	36.5	17	2	2	2	3	323
*Leontodon incanus* ssp. *tenuiflorus*	Rare	×	×	Asteraceae	19.7	37	23.9	1	2	2	2	63
*Linaria vulgaris*	Common	×	×	Plantaginaceae	62.5	33.2	36.7	1	2	4	3	640
*Lunaria rediviva*	Common		×	Brassicaceae	37.3	23	21.9	2	4	4	3	182
*Minuartia capillacea*	Rare	×	×	Caryophyllaceae	17.4	NA	8.7	1	1	2	2	6
*Origanum vulgare*	Common	×	×	Lamiaceae	54.2	22.2	16.8	1	2	3	3	992
*Papaver occidentale*	Rare		×	Papaveraceae	26.3	42.2	22.3	NA	NA	NA	NA	13
*Poa remota*	Rare	×	×	Poaceae	44.9	22.6	21.5	2	4	3	3	19
*Polycnemum majus*	Rare	×	×	Chenopodiaceae	35	NA	28.1	0	1	3	2	17
*Potentilla argentea*	Common		×	Rosaceae	13.2	43.9	18.5	1	2	2	2	302
*Potentilla multifida*	Rare	×	×	Rosaceae	22.6	25.5	16.4	2	3	4	2	8
*Prunella grandiflora*	Common		×	Lamiaceae	13.1	47.9	31.2	1	2	2	2	755
*Prunella laciniata*	Rare		×	Lamiaceae	16.6	43.7	26.7	1	2	2	1	72
*Rorippa islandica*	Rare	×	×	Brassicaceae	26.9	37.7	44.3	1	5	3	2	43
*Rumex hydrolapathum*	Rare	×	×	Polygonaceae	58.8	34.9	16.9	2	5	4	2	69
*Scrophularia auriculata*	Rare		×	Scrophulariaceae	61.3	37.7	37.4	2	5	4	2	12
*Scrophularia nodosa*	Common	×	×	Scrophulariaceae	80.1	30.6	26.9	2	4	4	2	801
*Scutellaria alpina*	Rare	×	×	Lamiaceae	25.3	36.5	27.6	1	2	2	3	19
*Selinum carvifolia*	Rare		×	Apiaceae	28.9	29.9	20.6	1	4	2	2	71
*Senecio halleri*	Rare	×	×	Asteraceae	6.8	39.8	18.8	1	2	2	2	31
*Sideritis hyssopifolia*	Rare	×	×	Lamiaceae	32.9	40.6	26.5	1	2	2	2	8
*Silene vallesia*	Rare		×	Caryophyllaceae	11.7	34.3	29.9	1	2	2	3	9
*Silene viscaria*	Rare		×	Caryophyllaceae	10.5	56.8	18.9	1	3	2	2	50
*Silene vulgaris*	Common		×	Caryophyllaceae	57.8	44.9	34.3	1	3	2	3	1305
*Taraxacum dissectum*	Rare	×	×	Asteraceae	15.9	NA	NA	1	2	4	2	8
*Tephroseris capitata*	Rare	×		Asteraceae	20.8	NA	NA	2	2	2	2	23
*Teucrium botrys*	Rare		×	Lamiaceae	21.2	21.9	28.9	0	2	2	2	134
*Teucrium chamaedrys*	Common		×	Lamiaceae	20.1	39.6	23.3	1	2	2	3	554
*Thlaspi rotundifolium* ssp. *corymbosum*	Rare	×		Brassicaceae	4.9	40.5	29.7	1	3	2	3	129
*Thlaspi sylvium*	Rare	×		Brassicaceae	4.5	41.3	19.6	1	4	2	2	5
*Trifolium repens*	Common	×	×	Fabaceae	48.8	32.6	32.6	1	3	4	3	1334
*Trifolium saxatile*	Rare		×	Fabaceae	11.9	38	18	0	1	2	1	18
*Veronica austriaca*	Rare		×	Plantaginaceae	26.9	39.9	34.8	1	2	2	2	14
*Veronica urticifolia*	Common		×	Plantaginaceae	39.6	36.5	22.3	2	4	3	2	628
*Xeranthemum inapertum*	Rare	×	×	Asteraceae	52	46.8	27.8	0	1	2	2	8

*Note*: Plant species included in the herbivore performance (Perf) and/or preference (Pref) experiments, with their rarity, plant family, mean plant size (cm), mean chlorophyll concentration in leaves (Chloro; in SPAD, which is a unit based on the leaf absorbance in red and near‐infrared, and is proportional to the chlorophyll amount present in the leaf; https://www.konicaminolta.com), mean SLA (mm^2^ mg^−1^), competitive strategy (Comp. strat.; can take a value between 1 and 4), indicator values for moisture (F), nutrients (N) and dominance in situ (D; can take a value between 1 and 5) and range size (highest number of 5 × 5 km grid squares occupied by a species during the last century in Switzerland; Bornand, 2014). All rare species, except *Bromus squarrosus*, have either a Swiss conservation priority (OFEV, 2019) or are near‐threatened or threatened (Swiss Red List of vascular plants; Bornand et al., 2016). *Bromus squarrosus* was considered rare after correspondence with A. Möhl from the National Data and Information Center of the Swiss Flora (https://www.infoflora.ch).

**TABLE A2:** Mean and standard deviation of the plant variables for regionally and locally rare and common plant species.

Rarity	Plant size	Chloro	SLA	Comp. strat.	F	N	D	Range size
Regional rarity								
Rare	25.47 ± 15.91	35.3 ± 11.55	26.19 ± 10.25	0.98 ± 0.57	2.59 ± 1.30	2.51 ± 0.84	2 ± 0.71	29.93 ± 30.60
Common	46.74 ± 21.31	33.3 ± 12.82	25.2 ± 7.63	1.2 ± 0.62	2.75 ± 0.79	3 ± 0.86	2.55 ± 0.51	839.15 ± 489.18
Local rarity								
Non‐dominant (D = 1)	24.34 ± 14.55	30.99 ± 12.81	32.68 ± 14.87	0.7 ± 0.48	2.7 ± 1.42	2.6 ± 0.97	‐	27.6 ± 23.82
Weakly dominant (D = 2)	31.5 ± 21.06	38.64 ± 10.47	25.13 ± 7.85	1.04 ± 0.67	2.64 ± 1.25	2.7 ± 0.84	‐	210.9 ± 329.38
Moderately dominant (D = 3)	37.62 ± 21.31	31.88 ± 12.38	24.34 ± 8.09	1.24 ± 0.44	2.62 ± 0.92	2.71 ± 0.90	‐	544 ± 632.38

*Note*: Plant variables are mean plant size (cm), mean chlorophyll concentration in leaves (Chloro; in SPAD, which is a unit based on the leaf absorbance in red and near‐infrared, and is proportional to the chlorophyll amount present in the leaf; https://www.konicaminolta.com), mean SLA (mm^2^ mg^−1^), competitive strategy (Comp. strat.; can take a value between 1 and 4), indicator values for moisture (F), nutrients (N) and dominance in situ (D; can take a value between 1 and 5 but our species have only values from 1 to 3) and range size (highest number of 5 × 5 km grid squares occupied by a species during the last century in Switzerland; Bornand, 2014).

**TABLE A3:** Experimental conditions.

Experiment	Plant species	Herbi.	Herbi. origin	Herbi. state	Actions	Exp. duration	Measure
Performance							
*Helix*	29	1	Park, Etis	Pre‐adult	Washing Body water saturation before and after Starving + gut standardisation	7 days	Final body weight
*Locusta*	23	1	Shop, Pocerias	Pre‐adult	Starving + gut standardisation	5 days	Final body weight
*Melolontha*	33	1	Field, Urmein	Second instar	Gut standardisation	15 days	Final body weight
*Spodoptera*	34						Mean weight of both experiments
1st experiment	33	2	Lab, Syngenta	4 days	Gut standardisation	10 days	Mean body weight
2nd experiment	28	3	Lab, Syngenta	7 days	Gut standardisation	5 days	Mean body weight
Preference							
*Helix*	54	1	Park, Etis	Pre‐adult		24 h	Goals per plant species
*Locusta*	49	1	Shop, Pocerias	Pre‐adult		Each 5 min for 30 min	Goals per plant species
*Melolontha*	55	1	Field, Bristen	Second instar	Potting plants 24 h before experiment	24 h	Goals per plant species
*Spodoptera*	55	3	Lab, Syngenta	10–12 days		24 h	Goals per plant species

*Note*: Summary of the experimental conditions for the assessment of the performance and preference of the four herbivore species. Plant species, Herbi. and Herbi. state refer to the number of plant species used per herbivore species, the number of herbivores used per experimental unit and the age or life‐stage of the herbivores when entering the experiments, respectively. Actions, Exp. duration and Measure refer to the plant and herbivore treatments, the time that a herbivore was allowed to feed on or choose a plant and the measure per herbivore species used as response variable in the analyses, respectively.

**TABLE A4:** Models for herbivore performance and preference.

Performance model	Preference model
**Herbivore final weight ~**	**Goals number ~**
**Initial plant size +**	**Initial mean plant size +**
**Herbivore initial weight +**	
**Herbivore species +**	**Herbivore species +**
**Herbivore species × Herbivore initial weight +**	
Range size (log) +	**Range size (log) +**
**N +**	N +
F +	F +
**SLA +**	SLA +
Chlorophyll +	Chlorophyll +
**Competitive strategy +**	Competitive strategy +
**Dominance +**	Dominance +
Range size (log) × Herbivore species +	**Range size (log) × Herbivore species +**
Range size (log) × N +	Range size (log) × N +
Range size (log) × F +	Range size (log) × F +
Range size (log) × SLA +	Range size (log) × SLA +
Range size (log) × Chlorophyll +	Range size (log) × Chlorophyll +
Range size (log) × Competitive strategy +	Range size (log) × Competitive strategy +
Range size (log) × Dominance +	Range size (log) × Dominance +
**Herbivore species × N +**	Herbivore species × N +
Herbivore species × F +	Herbivore species × F +
**Herbivore species × SLA +**	Herbivore species × SLA +
Herbivore species × Chlorophyll +	Herbivore species × Chlorophyll +
Herbivore species × Competitive strategy +	Herbivore species × Competitive strategy +
**Herbivore species × Dominance +**	Herbivore species × Dominance +
**1 | Plant species/Plant seed family**	**1 | Plant species**

*Note*: Performance and preference linear mixed‐effect models for all herbivore species together. Initial models (with all terms included) were reduced using a backward stepwise procedure and significant terms (in bold) were kept in the reduced models. Both response variables were centred an scaled per herbivore species and all covariates and explanatory variables, except herbivore species, plant species and plant seed family, were centred and scaled in the models.

**TABLE A5:** Phylogenetic test.

Variables	*K*	*p*
Plant size	0.125	.153
N	0.13	.132
F	0.111	.256
Dominance	0.111	.25
Competitive strategy	0.160	.051
Chlorophyll concentration	0.108	.38
SLA	0.182	.061
Range size	0.064	.835
Performance—Final weight		
*Helix aspersa maxima*	0.128	.671
*Locusta migratoria*	0.073	.832
*Melolontha melolontha*	0.180	.305
*Spodoptera littoralis*	0.139	.559
Preference—Goals		
*Helix aspersa maxima*	0.160	.109
*Locusta migratoria*	0.144	.297
*Melolontha melolontha*	0.144	.198
*Spodoptera littoralis*	0.134	.242

*Note*: Test for phylogenetic signal in the plant traits, indicator values, range size, performance (i.e. centred and scaled final weight) and preference (i.e. centred and scaled number of goals) of the four herbivore species. *K* is the test statistic and *p* is the corresponding *p* value.

**TABLE A6:** Performance model per herbivore species.

Performance—per herbivore species						
Herbivore species	*N*		SLA		Dominance	
	Estimate	*p*	Estimate	*p*	Estimate	*p*
Helix	0.017	.757	0.067	.155	−0.014	.770
Locusta	−0.142	.064	0.18	**.012**	0.345	**<.001**
Melolontha	−0.06	.49	−0.082	.205	−0.054	.393
Spodoptera	−0.148	.492	−0.07	.708	0.152	.406

*Note*: Estimates and corresponding *p* values of the herbivore‐specific linear mixed‐effect models testing for the effect of the plant variables significant in the performance model for all herbivore species on the performance of the herbivore species separately. Significant *p* values are highlighted in bold.

**TABLE A7:** Preference model per herbivore species.

Preference—per herbivore species		
Herbivore species	Range size (log)	
	Estimate	*p*
Helix	0.033	.823
Locusta	0.319	**.018**
Melolontha	−0.09	.572
Spodoptera	0.027	.861

*Note*: Estimates and corresponding *p* values of the herbivore‐specific linear models testing for the effect of the range size (log transformed; significant in the preference model for all herbivores species) on the preference of the herbivore species separately. Significant *p* values are highlighted in bold.
